# Sequential lateral positioning as a new lung recruitment maneuver: an exploratory study in early mechanically ventilated Covid-19 ARDS patients

**DOI:** 10.1186/s13613-022-00988-9

**Published:** 2022-02-12

**Authors:** Rollin Roldán, Shalim Rodriguez, Fernando Barriga, Mauro Tucci, Marcus Victor, Glasiele Alcala, Renán Villamonte, Fernando Suárez-Sipmann, Marcelo Amato, Laurent Brochard, Gerardo Tusman

**Affiliations:** 1 Laboratorio de Fisiología Experimental, Facultad de Medicina Humana, Universidad de Piura, Lima, Peru; 2 Intensive Care Unit, Hospital Rebagliati, Lima, Peru; 3 Laboratório de Pneumologia LIM–09, Disciplina de Pneumologia, Heart Institute (Incor) Hospital das Clínicas da Faculdade de Medicina da Universidade de São Paulo, São Paulo, Brazil; 4 Electronics Engineering, Aeronautics Institute of Technology, São Paulo, Brazil; 5 Intensive Care Unit, Hospital Universitario de La Princesa, Madrid, Spain; 6 Hedenstierna Laboratory, Surgical Sciences, Uppsala University, Uppsala, Sweden; 7 CIBER de Enfermedades Respiratorias, Instituto de Salud Carlos III, Madrid, Spain; 8 Keenan Research Centre, Li Ka Shing Knowledge Institute, St. Michael’s Hospital, Unity Health Toronto, 209 Victoria Street, Room 4-08, Toronto, ON M5B 1T8 Canada; 9 Interdepartmental Division of Critical Care Medicine, University of Toronto, Toronto, ON Canada; 10 Department of Anesthesiology, Hospital Privado de Comunidad, Mar del Plata, Argentina

**Keywords:** COVID-19, ARDS, PEEP, Postural lung recruitment

## Abstract

**Background:**

A sequential change in body position from supine-to-both lateral positions under constant ventilatory settings could be used as a postural recruitment maneuver in case of acute respiratory distress syndrome (ARDS), provided that sufficient positive end-expiratory pressure (PEEP) prevents derecruitment. This study aims to evaluate the feasibility and physiological effects of a sequential postural recruitment maneuver in early mechanically ventilated COVID-19 ARDS patients.

**Methods:**

A cohort of 15 patients receiving lung-protective mechanical ventilation in volume-controlled with PEEP based on recruitability were prospectively enrolled and evaluated in five sequentially applied positions for 30 min each: Supine-baseline; Lateral-1st side; 2nd Supine; Lateral-2nd side; Supine-final. PEEP level was selected using the recruitment-to-inflation ratio (R/I ratio) based on which patients received PEEP 12 cmH_2_O for R/I ratio ≤ 0.5 or PEEP 15 cmH_2_O for R/I ratio > 0.5. At the end of each period, we measured respiratory mechanics, arterial blood gases, lung ultrasound aeration, end-expiratory lung impedance (EELI), and regional distribution of ventilation and perfusion using electric impedance tomography (EIT).

**Results:**

Comparing supine baseline and final, respiratory compliance (29 ± 9 vs 32 ± 8 mL/cmH_2_O; *p* < 0.01) and PaO_2_/FIO_2_ ratio (138 ± 36 vs 164 ± 46 mmHg; *p* < 0.01) increased, while driving pressure (13 ± 2 vs 11 ± 2 cmH_2_O; *p* < 0.01) and lung ultrasound consolidation score decreased [5 (4–5) vs 2 (1–4); *p* < 0.01]. EELI decreased ventrally (218 ± 205 mL; *p* < 0.01) and increased dorsally (192 ± 475 mL; *p* = 0.02), while regional compliance increased in both ventral (11.5 ± 0.7 vs 12.9 ± 0.8 mL/cmH_2_O; *p* < 0.01) and dorsal regions (17.1 ± 1.8 vs 18.8 ± 1.8 mL/cmH_2_O; *p* < 0.01). Dorsal distribution of perfusion increased (64.8 ± 7.3% vs 66.3 ± 7.2%; *p* = 0.01).

**Conclusions:**

Without increasing airway pressure, a sequential postural recruitment maneuver improves global and regional respiratory mechanics and gas exchange along with a redistribution of EELI from ventral to dorsal lung areas and less consolidation.

*Trial registration* ClinicalTrials.gov, NCT04475068. Registered 17 July 2020, https://clinicaltrials.gov/ct2/show/NCT04475068

**Supplementary Information:**

The online version contains supplementary material available at 10.1186/s13613-022-00988-9.

## Introduction

Lung-protective ventilation has been the main supportive intervention in managing acute respiratory distress syndrome caused by SARS-CoV2 infection (C-ARDS), similar to ARDS from other causes. Due to the persistence of severe hypoxemia during C-ARDS, adjunctive measures such as prone positioning have been frequently used in addition to the limitation of tidal volumes, driving and plateau pressures, and the individual selection of positive end-expiratory pressure (PEEP) [1]. However, prone positioning needs trained personnel and is not without risks; complications such as accidental extubation, endotracheal tube obstruction or displacement, brachial plexus palsy, and facial and thoracic pressure ulcer have been described [2, 3].

Recruitment maneuvers are techniques designed to improve oxygenation by reopening and keeping open nonaerated parts of the lungs. The classical recruitment maneuvers are based on the application of high pressures, usually through a sustained inflation or stepwise increase of inspiratory pressure and/or of PEEP over a sufficient period of time. Clearly, they expose the patient to hemodynamic consequences [4, 5]. A recent randomized clinical trial using the stepwise approach described major complications of high pressures, and the arm using an open lung approach had higher mortality [6].

Lateral positioning does not require the application of higher pressures. However, it has mainly been attempted in unilateral pneumonia to improve oxygenation by improving the ventilation of the sick lung placed up [7, 8]. We reasoned that, in bilateral lung injury like C-ARDS, lateral positioning performed in sequential steps might act as a recruitment maneuver for each lung sequentially provided that sufficient PEEP is provided to prevent derecruitment. The different effects of the gravitational axis on each lung during lateral positioning can modify regional transpulmonary pressure (P_L_) that may help re-expand collapsed regions [9, 10]. This postural recruitment maneuver (P-RM) of the dependent parts of the lungs can be administered without changes in applied airway pressures or the need to turn the patient prone completely [11, 12].

We hypothesized that P-RM could be a useful adjuvant intervention improving lung aeration, helping to homogenize ventilation distribution without using high airway pressures or prone positioning. The objective of this study was to evaluate the feasibility and short-term physiological effects of the P-RM on pulmonary mechanics, gas exchange, lung aeration, and regional distribution of tidal ventilation and perfusion in patients with COVID-19-associated ARDS.

### Methods

A more detailed description of the methods is provided in the Additional file 1.

The study was approved by the Ethical Committee (Rebagliati Hospital, Lima, Perú, N° 1307) and registered at Clinicaltrials.gov NCT04475068. Informed consent was obtained from the legally authorized substitute decision-maker.

#### Patients

This single-center prospective observational study enrolled consecutively patients from July 2020 through Oct 2020. Inclusion criteria were: (1) patients with positive SARS-CoV-2 infection (confirmed by using real-time quantitative PCR on nasopharyngeal swabs); (2) moderate-to-severe ARDS as per the Berlin definition (PaO_2_/FiO_2_ ≦ 200 mmHg) under mechanical ventilation [13]; (3) Age ≧ 18 years old; (4) body mass index ≤ 35 kg/m^2^. Exclusion criteria were: (1) contraindications for EIT monitoring as (a) unstable spine or pelvic fractures; (b) pacemaker, automatic implantable cardio-defibrillator; (c) skin lesions between the 4th and 5th ribs where the EIT belt is positioned; (2) pregnancy; (3) mechanical ventilation > 1 week; (4) multi-organ failure; (4) hemodynamic instability defined as persistent mean arterial pressure lower than 60 mm Hg despite adequate fluid resuscitation and two vasopressors or increase of vasopressor dose by 30% in the previous 6 h; (5) COPD; (6) pneumothorax; and (7) increased intracranial pressure.

#### Mechanical ventilation settings

Patients were mechanically ventilated (Servo-I, Maquet), deeply sedated, and paralyzed. Patients were receiving volume-controlled ventilation, FIO_2_ adjusted to SpO_2_ 92–97%, tidal volume ≤ 6 mL/kg predicted body weight, adjusted to a plateau pressure of ≤ 28 cmH_2_O and a driving pressure ≤ 15 cmH_2_O, respiratory rate 20–30 breaths/min (adjusted to pH 7.20–7.40), an inspiratory–expiratory ratio of 1:1.5 to 1:2 (with an inspiratory pause of 10%) [14, 15]. The PEEP level was chosen using the one-breath decremental PEEP maneuver to calculate the recruitment-to-inflation ratio (R/I ratio) [16]: PEEP = 15 cmH_2_O for R/I ratio > 0.5, and PEEP = 12 cmH_2_O for R/I ratio ≤ 0.5. We reasoned that maintaining a sufficient PEEP level during the P-RM was important to avoid collapse during lateral position. Ventilatory settings were kept constant throughout the study.

#### Measurements

Regional ventilation (ΔZ = change in impedance) and aeration (EELI = end-expiratory lung impedance) were obtained with an EIT monitor (Enlight 1800, Timpel, Brazil). The distribution of tidal ventilation was determined as a percentage of regional ΔZ/total ΔZ and used to estimate regional tidal volume (VT_r_ = regional ΔZ/total ΔZ × total V_T_). Regional lung compliance was calculated as regional VT_r_/ΔP. The change in lung aeration was estimated by the change in EELI [ΔEELI × (V_T_/ΔZ)]. Lung perfusion was obtained by injecting a 10-mL bolus of 7.5% hypertonic saline solution into a central venous catheter during an expiratory pause [17].

**Fig. 1:**
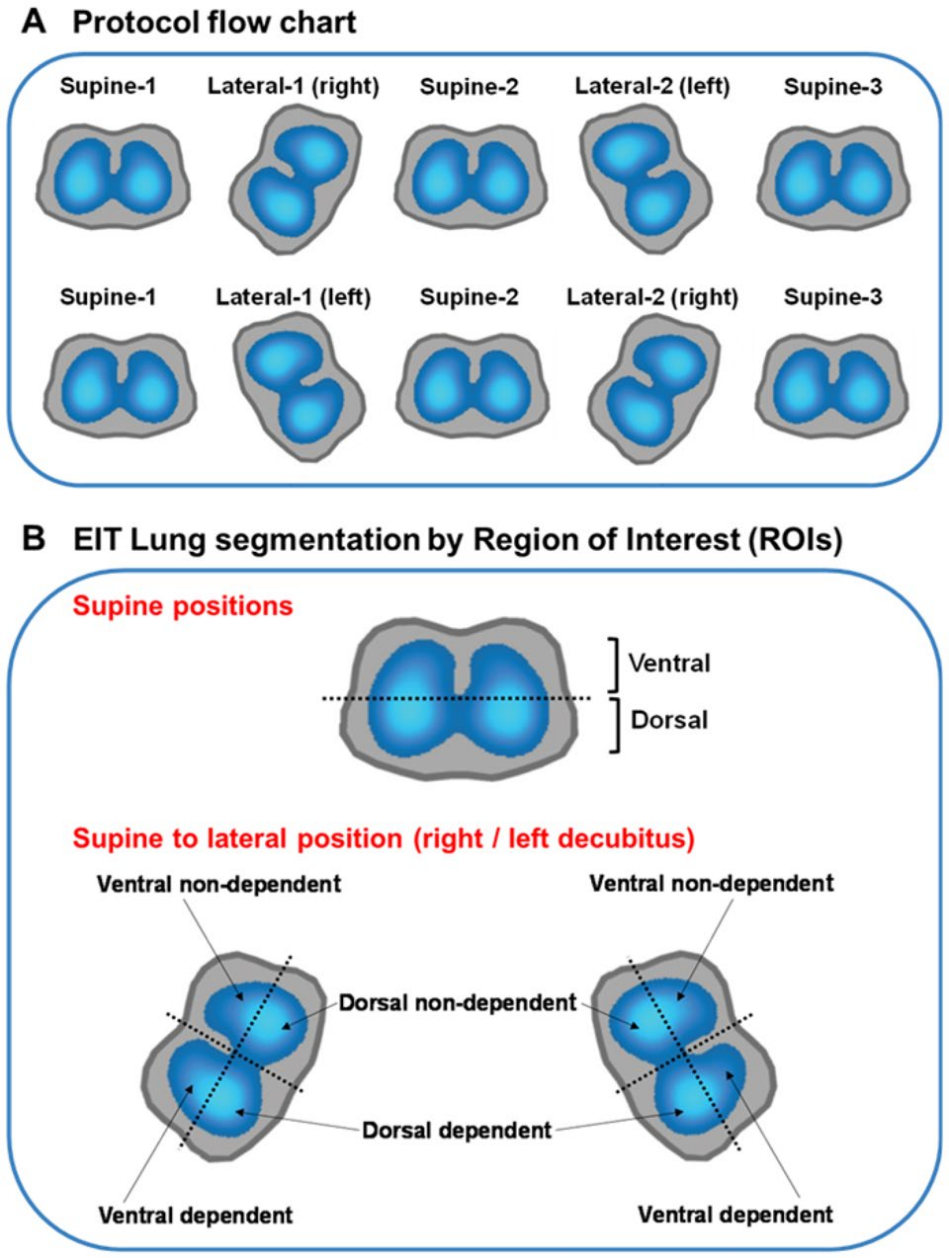
Protocol flowchart and EIT lung segmentation. The protocol flowchart (**A**) is shown at the top. The positioning sequence begins with the less ventilated lung evaluated by EIT positioned upwards in L1. In section **B** is shown the EIT lung segmentation by ROIs. To compare changes during supine position, the lungs were segmented into two equally sized ROIs: ventral and dorsal. To compare changes from supine to lateral position, the lungs positioned upwards (non-dependent) or downwards (dependent lung) during L1 or L2, were segmented into four ROIs or quadrants: ventral non-dependent, dorsal non-dependent, dorsal dependent, and ventral dependent. EIT: electrical impedance tomography; ROI: region of interest; L1: first lateral; L2: second lateral

Ventilation and perfusion maps were segmented into regions of interest (ROI) [18] (Fig. 1). To compare the changes between position steps, the lungs were segmented into two equally sized ROIs: ventral (upper lung or non-dependent half) and dorsal (lower lung or dependent half); we further divided the lungs into four ROIs for the lateral position according to the new situation when lateralized (ventral dependent or non-dependent, dorsal dependent or non-dependent).

Pleural pressure was estimated by esophageal manometry (Cooper Surgical). Pulmonary mechanics was measured during inspiratory and expiratory holds of 0.5 and 4 s, respectively [15].

Lung aeration was assessed by lung ultrasound (MyLab Gold 25, Esaote) using the lung ultrasound aeration score (LUS) calculated by summing regional scores (0–3 points) obtained in 6 regions of each lung [19] and the consolidation score to assess the degree of juxta-pleural consolidation; each explored area was divided into four grades and scored between 0 and 3 [20].

#### Protocol

Patients were studied in five body positions in sequential order, each maintained during 30 min: Supine-1 (S1), which served as the baseline condition; Lateral-1 (L1-the less ventilated lung evaluated by EIT was positioned up first); Supine-2 (S2-after first lateral position); Lateral-2 (L2-the contralateral lung was positioned up); Supine-3 (S3-after second lateral position) (Fig. 1). Lateral positioning was done with an inclination of 30° using a custom-made support cushion lined with a special foam (see Additional file 1: Fig. S1).

At the end of each 30-min period, arterial blood gas samples, hemodynamics, pulmonary mechanics, and both EIT and lung ultrasound images were recorded. In one patient, it was only possible to obtain ultrasound and perfusion images at S1 and S3, and it was not possible to also insert an esophageal balloon.

### Statistical analysis

Due to the lack of previous comparable studies on the subject that allow the calculation of a sample size, we initially chose a convenience sample of 12 patients and increased it to 15 patients after obtaining additional EIT equipment from Timpel. Descriptive statistics were expressed as mean (standard deviation), median (interquartile range), or counts and percentages, as appropriate. Normality was assessed by the Shapiro–Wilk test. Differences between measurements at different body positions were evaluated using a restricted maximum likelihood analysis for the mixed-effects model. When multiple comparisons were made, P values were adjusted through Sidak post hoc correction. The PR-M effects are expressed as mean difference and 95% CI or median difference (interquartile range). A paired t-test or Wilcoxon signed-rank test was used to analyze paired differences between two positions, as appropriate. All tests were 2-tailed, and differences were considered significant when P-value < 0.05. Analysis was performed using Prism version 8 (GraphPad Software).

## Results

Fifteen patients with moderate-to-severe C-ARDS were enrolled. Median ventilation days before enrollment was 0.8 (0.3–2.1). Eight patients had an R/I ratio higher than 0.5 and were ventilated with a PEEP of 15 cmH_2_O. Six patients began the lateral positioning sequence in the left (right lung up) and nine patients in the right decubitus position (left lung up). Patients' characteristics are summarized in Table 1.

**Table 1:** Demographic, respiratory and hemodynamics data

Demographic data	
Number of patients, n	15
Age, years	53 (50–62)
Male, n (%)	14 (93)
BMI, kg/m^2^	27 (24–29)
APACHE II, Score	13 (11–16)
Recruitment-to-Inflation Ratio > 0.5, n (%)	8 (53)
Number of days from the symptom’s onset to intubation	14 (11–16)
Ventilation days before enrollment	0.8 (0.3–2.1)
Death in the ICU, n (%)	6 (40)

	Sequential steps				
	Supine-1	Supine-2	Supine-3	*N*	*p*
Respiratory parameters					
Driving airway pressure, cmH_2_O	12.5 ± 2.4	11.6 ± 2.2	11.2 ± 2.1	15	< 0.01
Respiratory system compliance, mL/cmH_2_O	28.5 ± 8.5	30.6 ± 8.1	31.6 ± 8.4	15	< 0.01
Plateau airway pressure, cmH_2_O	26.8 ± 2.9	25.9 ± 2.7	25.6 ± 2.5	15	< 0.01
Peak airway pressure, cmH_2_O	32.4 ± 3.1	31.8 ± 3.3	31.3 ± 2.8	15	< 0.01
Mean airway pressure, cmH_2_O	19.3 ± 1.8	19.1 ± 1.8	19.0 ± 1.7	15	< 0.05
Tidal volume, mL/kg	5.4 ± 0.6	5.4 ± 0.6	5.4 ± 0.6	15	0.11
Expiratory minute volume, L/min	8.5 ± 1.1	8.6 ± 1.1	8.6 ± 1.1	15	0.12
Total PEEP, cmH_2_O	15.5 (12.5–15.8)	15.4 (12.4–16.1)	15.7 (12.7–15.9)	15	0.55
Resistance, cmH_2_O/L/min	10.8 ± 1.9	11.3 ± 2.1	11.0 ± 2.0	15	0.38
Driving transpulmonary pressure, cmH_2_O	10.9 ± 2.5	10.2 ± 2.3	9.7 ± 2.1	14	< 0.01
Lung compliance, mL/cmH_2_O	33.8 ± 10.9	35.7 ± 10.5	37.5 ± 10.5	14	0.01
Inspiratory transpulmonary pressure, cmH_2_O	15.9 ± 4.7	15.1 ± 4.5	14.7 ± 3.7	14	0.08
Expiratory transpulmonary pressure, cmH_2_O	5.0 ± 3.6	4.9 ± 4.1	5.1 ± 3.1	14	0.8
Chest wall compliance, mL/cmH_2_O	245 (188–262)	239 (183–270)	240 (198–291)	14	0.93
Gas exchange					
PaO_2_/FIO_2_, mmHg	137.5 ± 36.3	158.4 ± 35.9	163.8 ± 46.1	15	< 0.01
PaCO_2_, mmHg	63 (58–75)	64 (52–71)	63 (55–81)	15	0.08
pH	7.28 ± 0.1	7.28 ± 0.11	7.27 ± 0.1	15	0.61
SpO_2_ (%)	96 (95–98)	98 (96–99)	98 (96–99)	15	0.05
PaCO_2_–ETCO_2_	15.8 ± 12.1	15.4 ± 13.4	15.8 ± 12.9	15	0.9
Hemodynamics					
Heart rate, beats/minute	92 ± 18	90 ± 17	90 ± 18	15	0.64
Systolic arterial pressure, mmHg	127 ± 16	124 ± 19	123 ± 19	15	0.76
Diastolic arterial pressure, mmHg	68 ± 8	68 ± 11	69 ± 8	15	0.91
Mean arterial pressure, mmHg	88 ± 10	87 ± 13	88 ± 11	15	0.89

BMI: body mass index; PBW: predicted body weight. PEEP: positive end-expiratory pressure; PaO_2_/FIO_2_: partial pressure of oxygen in arterial blood/inspired oxygen fraction ratio; PaCO_2_: partial pressure of carbon dioxide in arterial blood; SpO_2_: oxygen saturation; ETCO_2_: end-tidal CO_2_; *n*: number of patients

Continuous variables are shown as mean ± SD or median (IQR) based on their distribution. Mixed model was used to compare the periods in supine position

### Comparisons between baseline and final supine positions

#### Lung mechanics and gas exchange

Respiratory system and lung compliance increased by 3.2 mL/cmH_2_O (95% CI 1.5–4.8; *p* < 0.001) and 3.7 mL/cmH_2_O (95% CI 0.9–6.5; *p* = 0.01), respectively; driving pressure and transpulmonary driving pressure decreased by 1.3 cmH_2_O (95% CI − 2.0 to − 0.7; *p* < 0.001) and 1.2 cmH_2_O (95% CI − 1.9 to − 0.6; *p* < 0.001), respectively. Oxygenation (PaO_2_/FIO_2_) improved by 26.3 mmHg (95% CI 7.9–44.8; *p* < 0.001) (Table 1).

#### EIT measurements

**Fig. 2:**
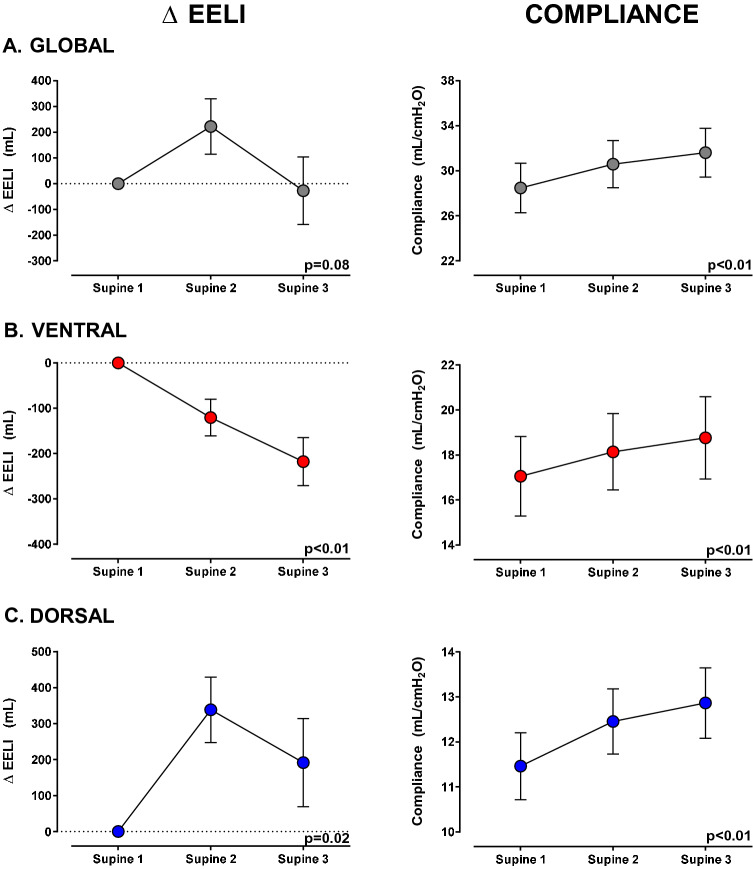
End-expiratory lung impedance and compliance changes during supine position steps, from supine-1 (baseline) to supine-3 (after second lateral positioning), segmenting the lung into ventral and dorsal areas. The left column shows changes in the EELI, and the right column shows changes in compliance. The figures on the upper panel **A** show global lung changes, while the figures in the middle panel **B** show changes in the ventral (anterior half) part of the lung, and the figures on the bottom panel **C** show changes in the dorsal (posterior half) of the lung. The left column shows that global EELI did not change between supine-1 and supine-3, but the EELI decreased in the ventral region and increased in the dorsal region. This redistribution of EELI was accompanied by a progressive increase of the global and regional compliance (both ventral and dorsal) (right column). Δ EELI (mL): end-expiratory lung impedance change. Data are shown as mean ± SEM (standard error of mean). Mixed model was used for statistical analysis

Regional compliance (C_Z_) increased in both the ventral and dorsal regions: ventral by 1.4 mL/cmH_2_O (95% CI 0.6–2.2; *p* = 0.002) and dorsal by 1.7 mL/cmH_2_O (95% CI 0.3–3.2; *p* = 0.03) (Table 1, Fig. 2). The global end-expiratory lung impedance (EELI) did not change, but EELI decreased in the ventral lung region by 218 mL (95% CI − 331 to − 104; *p* < 0.001) and increased in the dorsal lung region by 192 mL (95% CI − 72 to 455; *p* = 0.02) (Fig. 2 and Additional file 1: Table S1).

The ventilation distribution was predominantly dorsal and did not change, while the percentage of regional distribution of perfusion slightly increased in the dorsal region (*p* = 0.01) (Additional file 1: Table S1).

#### Lung ultrasound

**Fig. 3:**
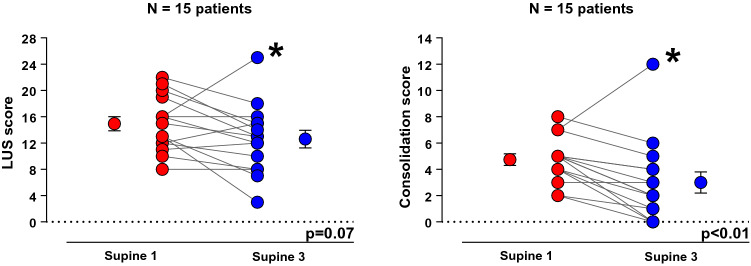
Evaluation of aeration by lung ultrasound between supine-1 (baseline) and supine-3 (after second lateral positioning). The analysis of the individual data shows an improvement in LUS score (left figure) and consolidation score (right figure) in the majority of patients. A non-responder patient is identified with asterisk *LUS score: lung ultrasound score. Paired t test was used for the analysis of LUS score and Wilcoxon signed-rank test for the consolidation score

The consolidation score decreased [5 (4–5) vs. 2 (1–4), *p* < 0.01] while the global LUS score did not significantly change (14.9 ± 4.2 vs. 12.6 ± 5.2, *p* = 0.07) (Fig. 3 and Additional file 1: Table E1). One patient presented a decrease in aeration after lateral positioning sequence on ultrasound. When excluding this patient from the analysis, LUS significantly decreased (*p* < 0.01) (Additional file 1: Fig. S2; Additional file 2: see Video S1 Additional file 3: and Video S2).

### Changes during the lateral position compared to supine

#### Lung mechanics and gas exchange

From the supine baseline to Lateral-1 as well as from the second supine position to Lateral-2, respiratory system compliance decreased (*p* < 0.01), reflecting a decrease in chest wall compliance by 53 mL/cmH_2_O (95% CI − 90 to − 16; *p* < 0.01) without changes in lung compliance (*p* > 0.05). Oxygenation (PaO_2_/FIO_2_) did not change (*p* > 0.05) (Additional file 1: Table S2).

#### EIT measurements

**Fig. 4:**
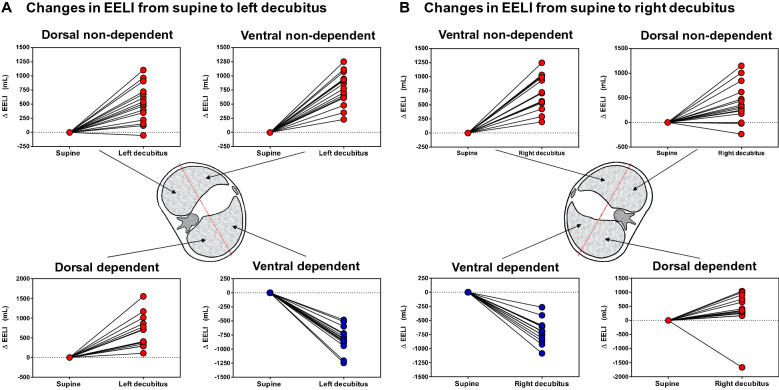
Changes in the end-expiratory lung impedance when going from supine to lateral position. Changes in end-expiratory lung impedance are presented, segmenting the lung into four quadrants from supine to left decubitus (left panel, **A**) and from supine to right decubitus (right panel, **B**). Regardless of the lateralized side, it is observed a decrease of EELI in the ventral quadrant of the lung placed down (dependent lung) and a significant increase in the other three quadrants. Δ EELI = End-expiratory lung impedance change. Data are shown as mean ± SEM

For comparing changes in EELI occurring during lateral positions (left or right decubitus), the lung was segmented into four quadrants (Fig. 1). EELI increased in both non-dependent uppermost quadrants (ventral and dorsal) (*p* < 0.001); in the dependent (lowermost) lung, EELI increased in the dorsal quadrant (*p* < 0.001) and decreased in the ventral quadrant (*p* < 0.001) (Fig. 4).

EIT-aeration analysis was performed. When changing from baseline supine to either Lateral-1 and Lateral-2: EELI increased in the non-dependent lung by 1222 ± 422 mL and 1109 ± 621 mL, respectively (*p* < 0.001); and remained unchanged in the dependent lung by − 22 ± 409 mL and − 68 ± 357 mL, respectively (*p* > 0.9) (Additional file 1: Fig. S3). Regional compliance in the non-dependent lung decreased in both Lateral-1 and Lateral-2 positions (*p* < 0.001), while the dependent lung did not significantly change (Additional file 1: Table S2).

When moving from supine to either Lateral-1 and Lateral-2, the regional distribution of ventilation decreased in the non-dependent lung by 13% and 18% (*p* < 0.001), respectively; and increased in the dependent lung in the same proportion, while the distribution of perfusion did not change (Additional file 1: Table S2).

When returning to supine positions (Supine-2 or Supine-3), the regional compliance of the lung previously positioned upwards (non-dependent lung) during lateralization increased (*p* < 0.01). At the same time, the lung previously placed downwards (dependent lung) maintained its compliance. At the supine-final step (Supine-3), both lungs improved their compliance compared to baseline-supine (see Additional file 1: Figs. S4 and S5).

#### Lung ultrasound

Both the LUS and consolidation scores improved in the non-dependent lung during lateral positioning (*p* < 0.001) (Additional file 1: Table S2). The dependent lung could not be evaluated by ultrasonography for technical reasons.

No changes in arterial pressure, heart rate, and SpO_2_ were observed throughout the protocol. An increase in PaCO_2_ and PaCO_2_–ETCO_2_ gradient was observed when moving from Supine-2 to Lateral-2 positions (Table 1 and Additional file 1: Table S2).

Finally, we compared patients with low recruitability (R/I ratio ≤ 0.5) and high recruitability (R/I ratio > 0.5) (see Additional file 1: Table S3). We observed in both groups the same trends for a decrease in driving pressure and driving transpulmonary pressure, an increase in respiratory system compliance and lung compliance along with an increase of PaO_2_/FIO_2_ ratio and a decreased in LUS score and consolidation score. However, these changes were only significant in the high recruitability group. The EIT analysis showed in both groups a similar trend for an increase in the ventral and dorsal compliance along with a decrease of EELI in the ventral lung region, but these changes were only significant in the high recruitability group. The changes in EELI of the dorsal lung region were not significant in both groups.

## Discussion

The main findings of this study can be summarized as follows. First, we found that this postural recruitment maneuver was effective and resulted in a lung re-expansion that was maintained at least 30 min after returning to the supine position as indicated by the improvement in the ultrasound consolidation score along with a redistribution of EELI measured by EIT; an improvement in global and regional respiratory mechanics; and a better oxygenation. Second, these benefits were achieved without the need to pressurize the patient's lungs, and the positional changes were well tolerated in all patients in terms of hemodynamic and gas exchange stability.

In the lateral position, the upper lung is submitted to a larger P_L_ than the lower lung due to the reduction in pleural pressure thanks to the new vertical gravitational pressure gradient [9–11]. By altering the gravitational gradient of pleural pressure with position, lateral positioning can conveniently alter regional P_L_ while keeping the same applied airway pressure. This leads to higher P_L_ in the non-dependent lung and lower P_L_ in the dependent one, mainly because the lateral axis is longer than the anteroposterior, promoting re-expansion of collapsed lung regions in the uppermost lung [9–11, 21]. If the new P_L_ succeeds in opening collapsed lung regions in the uppermost lung according to Laplace's law and lung hysteresis, these regions will remain open when changing to the opposite position, as long as sufficient PEEP is applied to maintain those regions open and minimize the risk of collapse of the dependent lung in the lateral position [22, 23].

The regional analysis in the lateral position suggests the occurrence of recruitment and additional inflation of the non-dependent lung. In contrast, the dependent lung did not induce any derecruitment (except in one patient) during lateral positioning. An increase in PaCO_2_ and PaCO_2_–ETCO_2_ gradient was observed during the second lateral positioning, suggesting regional overinflation.

Initial CT-scan based reports described different phenotypes of C-ARDS with similar hypoxemia severity and suggested using the response to PEEP to differentiate these phenotypes and guide PEEP selection [24, 25]. We set PEEP based on lung recruitability using the recruitment-to-inflation ratio, which evaluates the change in end-expiratory lung volume by changing PEEP and comparing the compliance of the recruited lung with the compliance of the baby lung [10]. Based on this approach, ultrasound analysis comparing baseline and final supine positions showed an aeration improvement in most patients but different depending on the degree of recruitability.

Interestingly, the analysis of the lungs divided by quadrants in the lateral position, regardless of the lateralized side, showed an increase of EELI in all the non-dependent lungs (expected by the increase in transpulmonary pressure). In contrast, the two quadrants of the dependent lung showed changes of EELI in opposite directions, with an increase in the dorsal quadrant and a decrease in the ventral. A combination of effects could explain these findings in the dependent lung: an increase in the diameter in the lateral axis with respect to the anteroposterior [10], the overlying weight of the heart and mediastinum, and the limitation of thoracic expansion in the lateral position (evidenced by the observed decrease of respiratory system compliance due to a decrease in chest wall compliance) [26, 27]. In the prone position, it has been hypothesized that the decrease in C_cw_ results in a better distribution of the tidal volume, more towards the dependent part of the lung [28, 29], and these benefits may be maintained after returning to supine position [30].

In our study, when the patients returned to the supine position, it was observed similarly a redistribution of EELI characterized by a decrease of EELI in the ventral part of the lung and an increase in the dorsal lung region associated with fewer consolidations in ultrasound. Furthermore, the regional compliance of the lung that was positioned upwards during lateral positioning increased. As a result, the regional and global respiratory mechanics and oxygenation improved at the end of the lateral positioning sequence (Additional file 1: Fig. S5).

Our findings are in line with previous studies comparing global and regional parameters between supine and prone positions. Pelosi et al. [29] found an increase in respiratory system compliance mainly due to an increase of the lung compliance, but without changes in the global end-expiratory lung volume, after prone patients returned to the supine position. Katira et al. [31] described in a lung injury model that prone positioning reduced the vertical pleural pressure gradient and homogenized regional ventilation and compliance between the dependent and non-dependent regions. The changes in lung shape with prone position (extension in the cephalocaudal axis) possibly explained these homogenizing effects. If similar effects occur in the lateral position, they deserve further investigation.

We also observed an increase in the percentage of regional distribution of perfusion in the dorsal lung, maybe related to the increase in EELI in that region. However, there was no redistribution of the proportion of regional tidal ventilation, which was already proportionally greater in the dorsal part of the lung at baseline.

The decrease in driving pressure, which has been associated with mortality [32], was statistically significant although small in absolute terms (1.3 cmH_2_O), which could be questioned in terms of its clinical relevance. However, it should be interpreted in the context of patients already managed with a lung-protective strategy that included low V_T_ (5.4 mL/kg PBW) and plateau pressure (26.8 cmH_2_O), relatively high levels of PEEP (12.5–15.8 cmH_2_O), and a low baseline driving pressure (12.5 cmH_2_O).

Interestingly, an exploratory analysis showed a greater benefit from applying the P-RM in the subgroup of patients with high potential for lung recruitment, which highlights the clinical relevance of classifying patients based on this approach.

There are several limitations in the present study. We included a small number of patients and must thus be considered an exploratory and descriptive study. We enrolled early mechanically ventilated C-ARDS patients in whom application of a P-RM could be more effective [33]. We used a sequential order rather than a randomized order starting with the less ventilated lung up based on EIT for greater patient safety. Lung ultrasound is operator-dependent technique and those responsible for the acquisitions of the images (SR and FB) were not blinded to body positioning. The individualization of PEEP is essential to avoid dependent lung collapse during lateral positioning and to keep open the non-dependent lung when returning to the supine position. For practical purposes, we only used two levels of PEEP (12 or 15 cmH_2_O) based on recruitability assessed by recruitment-to-inflation ratio, however, we must highlight that C-ARDS shows differences compared with other types of ARDS, especially at early phase [24, 25]. Furthermore, the PEEP selection must be adapted not only to the evolution of the disease, but also to patient’s characteristics, like obesity [26]. The optimum ventilator settings during the P-RM are unknown and should be tested in future studies. Changes were assessed after 30 min, but the optimal duration of the maneuver is unknown, and thus, merits further study as well as testing the duration of the benefits observed. We also do not know the clinical impact of applying a postural recruitment maneuver on unilateral lung diseases. Therefore, caution is necessary when extrapolating the current data. Despite these limitations and putting all our findings together, the P-RM was effective in recruiting the lungs when they were in the non-dependent uppermost position, while no derecruitment occurred when they were in the dependent lowermost position. The PEEP level used in the studied patients was relatively high, and this contributed to avoiding derecruitment in all positions. This suggests that P-RM may contribute to enhancing lung protection in a synergistic way.

### Conclusions

Applying a sequential postural recruitment maneuver in moderate-to-severe C-ARDS patients improves global and regional respiratory mechanics and oxygenation and promotes a redistribution of EELI from ventral to dorsal areas with less consolidations. Lateral positioning performed in sequential steps can act as a recruitment maneuver without increasing airway pressure and negative hemodynamic consequences. However, until the long-term effects of this maneuver are known, this procedure should not be considered an alternative to the prone positioning.

## Supplementary Information

**Additional file 1.** Sequential lateral positioning as a new lung recruitment maneuver: an exploratory study in early mechanically ventilated Covid-19 ARDS patients.

**Additional file 2.** Lung ultrasound images of a responder patient throuthgout lateral positioning sequence.

**Additional file 3.** Lung ultrasound images of a non-responder patient throuthgout lateral positioning sequence.

## Data Availability

Dataset is available upon reasonable request to Dr. Rollin Roldán (rollinroldan@yahoo.es).

## Abbreviations

C-ARDS: Acute respiratory distress syndrome caused by SARS-CoV2 infection
PEEP: Positive end-expiratory pressure
P_L_: Transpulmonary pressure
P-RM: Postural recruitment maneuver
R/I ratio: Recruitment-to-inflation ratio
EIT: Electric impedance tomography
ΔZ: Change in impedance
EELI: End-expiratory lung impedance
C_Z_: Regional lung compliance
ROI: Region of interest
LUS score: Lung ultrasound score
PaO_2_/FIO_2_: Partial pressure of oxygen in arterial blood/inspired oxygen fraction ratio
SpO_2_: Oxygen saturation
PaCO_2_: Partial pressure of carbon dioxide in arterial blood
ETCO_2_: End-tidal CO_2_
C_cw_: Chest wall compliance
