# Supplementary material for: Sequential lateral positioning as a new lung recruitment maneuver: an exploratory study in early mechanically ventilated Covid-19 ARDS patients
Source: Ann Intensive Care. 2022 Feb 12;12:13. doi: 10.1186/s13613-022-00988-9 (PMC8840950; doi:10.1186/s13613-022-00988-9)
Supplement: Supplementary file 1 — Additional file 1. Sequential lateral positioning as a new lung recruitment maneuver: an exploratory study in early mechanically ventilated Covid-19 ARDS patients. [file 13613_2022_988_MOESM1_ESM.docx]

**Sequential lateral positioning as a new lung recruitment maneuver: an exploratory study in early mechanically ventilated Covid-19 ARDS patients**

Rollin Roldán, MD^1,2,3^, Shalim Rodriguez, MD^1,2^, Fernando Barriga, MD^1,2^, Mauro Tucci, MD^3^, Marcus Victor, Ph.D^3,4^, Glasiele Alcala, RRT^3^, Renán Villamonte, MD^1,2^, Fernando Suárez-Sipmann, MD^5,6,7^, Marcelo Amato, MD^3^, Laurent Brochard, MD^8,9^, Gerardo Tusman, MD^10^.

**ADDITIONAL FILE**

**Methods**

The study was performed from July 2020 through Oct 2020 at Hospital Rebagliati, Lima, Perú. This study was approved by the Ethics Committee for Clinical Studies (Hospital Rebagliati, N° 1307; Clinicaltrials.gov NCT04475068). Informed consent was obtained from the legally authorized substitute decision-maker.

**Inclusion criteria**

1. Patients with positive SARS-CoV-2 infection ^(*)^
2. Patients with moderate-to-severe ARDS as per the Berlin definition (PaO_2_/FiO_2_ ≦ 200 mmHg) under mechanical ventilation [1]
3. Age ≧ 18 years old
4. Body mass index ≤ 35 kg/m2

*(*) Positive SARS-CoV-2 infection was defined as being positive in RT-PCR (real-time reverse transcriptase-polymerase chain reaction) assay using nasal or pharyngeal swab samples.*

**Exclusion criteria**

1. Contraindications for EIT monitoring:
   1. Unstable spine or pelvic fractures
   2. Pacemaker, automatic implantable cardio-defibrillator
   3. Skin lesions between the 4th and 5th ribs where the EIT belt is positioned
2. Pregnancy.
3. Mechanical ventilation > 1 week.
4. Multi-organ failure.
5. Hemodynamic instability defined as persistent mean arterial pressure lower than 60 mm Hg despite adequate fluid resuscitation and two vasopressors or increase of vasopressor dose by 30% in the previous 6 hours.
6. COPD.
7. Pneumothorax.
8. Increased intracranial pressure.

**Sedation, ventilation, and hemodynamic management**

Eligible patients were identified for researchers by daily screening in the intensive care unit. Patients received profound sedation before the protocol (Richmond Agitation Sedation Scale - RASS -5) with a continuous infusion of propofol 80 μg/kg/min and fentanyl 100 μg/h. The level of sedation was quantitatively evaluated by using Bispectral index (BIS, Aspect Medical Systems, Norwood, USA) ≦ 60 (corresponding to Richmond Agitation Sedation Scale - RASS -5). After baseline measurement and before the P-RM, if not previously on neuromuscular blockers therapy, patients received a bolus of rocuronium of 50 mg followed by an infusion of 7-9 μg/kg/min monitored by the train-of-four for the duration of the protocol. Paralysis was confirmed with a train-of-four count (TOF count) of zero.

Patients were mechanically ventilated (Servo-I, Maquet, Solna, Sweden) as follows:

- Volume controlled ventilation
- FIO_2_ adjusted to SpO_2_ 92-97%
- Tidal volume ≤ 6ml/kg predicted body weight ^(**)^ (adjusted to plateau pressure ≤ 28 cmH_2_O and driving pressure ≤ 15 cmH_2_O)
- Respiratory rate= 20-30 breaths/min (adjust to pH 7.20-7.40)
- Inspiratory-expiratory ratio of 1:1.5 to 1:2 (with an inspiratory pause of 10%)

*(**) Predicted body weight calculation: Male: 50 + 0.91 × (height in cms-152.4) kg. Female: 45.5 + 0.91 x (height in cms-152.4) kg*

PEEP level was chosen using the one-breath decremental PEEP maneuver to calculate the Recruitment-to-Inflation Ratio (R/I ratio) [2]. The maneuver consists of an abrupt decrease of PEEP, from 15 to 5 cmH_2_O, to assess the lung volume and compliance differences. The R/I ratio > 0.5 predicts patients with a high potential for lung recruitability. R/I ratio was calculated as follows (please refer to <https://rtmaven.com/> for instructional videos):

R/I ratio = $\frac{VTe, PEEP15\to5 - VTe, PEEP15}{\mathrm{PEEP}15-PEEP5}$ × $\frac{Pplat, PEEP5 - PEEP5}{VTe, PEEP5}$ – 1

V_Te,PEEP15→5_ : Expiratory tidal volume measured at the time of changing PEEP 15 to 5

V_Te,PEEP15_ : Expiratory tidal volume measured at the time of PEEP 15

V_Te,PEEP5_ : Expiratory tidal volume measured at the time of PEEP 5

P_plat,PEEP5_ : Plateau pressure at the time of PEEP5

According to the result of such test, patients with R/I > 0.5 received 15 cmH_2_O of PEEP while patients with R/I ≤ 0.5 were ventilated with 12 cmH_2_O of PEEP along the protocol.

**Respiratory mechanics**

An esophageal balloon catheter (Ackradt esophageal balloon, Cooper Surgical, Trumbull, CT). was inserted and filled with 1.0 mL as a minimal non-stress volume. Proper catheter position was ensured by a positive pressure occlusion test using a chest compression maneuver (i.e., correct positioning assumed where the slope of the regression between pressure changes in the balloon vs. airway: 1.0±0.2, during proximal airway occlusion) [3], and this was confirmed in all cases. Airway flow and pressure sensors were connected to the respiratory circuit at the airway opening proximal to the Y-piece. We used a customized software to analyze respiratory mechanics (flow, Paw, and Pes values) with a program running in Labview (National Instruments, USA).

The following respiratory mechanics parameters were analyzed:

- Plateau pressure (P_plat_) = airway pressure at end-inspiratory pause (0.5sec).
- Total PEEP = airway pressure at end-expiratory pause (4sec).
- Driving pressure (ΔP) = Pplat – total PEEP
- Transpulmonary pressure (P_L_) = P_ao_ - P_es_
- End-inspiratory P_L_ (P_Linsp_) = P_plat_ - P_es_ at end-inspiratory pause
- End-expiratory P_L_ (P_Lexp_) = PEEP - P_es_ at end-expiratory pause
- Transpulmonary driving pressure (ΔP_L_) = end-inspiratory P_L_ - end-expiratory P_L_
- Delta esophageal pressure (ΔP_es_) = P_es_ at end-inspiratory pause – P_es_ at end-expiratory pause
- Respiratory-system compliance (C_rs_) = V_T_ / ΔP
- Lung compliance (C_Lung_) = V_T_ / ΔP_L_
- Chest wall compliance (C_cw_) = V_T_ / ΔP_es_
- Inspiratory airway resistance [(Raw = Ppeak-Pplat /inspiratory flow)]

**Electrical impedance tomography (EIT)**

A belt containing 32 electrodes was placed around the thorax at the level of the 4^th^-5^th^ intercostal space and connected to the EIT monitor (Enlight 1800, Timpel, São Paulo, Brasil). The integrated and synchronized pressure and flow sensor of the Enlight monitor was connected to the proximal airway. EIT data were generated by the application of electrical currents (5mA;125kHz), with measurement of the voltage differences between neighboring electrode pairs in a sequential rotating pattern. High-resolution functional images of ventilation and perfusion were sampled at a rate of 50 Hz and represented in a 32 x 32 pixels field. A scan displaying the ventilation-induced impedance changes (ΔZ) was obtained every 50ms.

The following EIT-derived parameters were calculated:

- Delta Z (∆Z): variation of impedance during a tidal breath.
- Delta end-expiratory lung impedance (∆EELI): variation of baseline impedance plethysmography used as a surrogate of change in End-expiratory lung volume.
- Distribution of regional tidal ventilation was determined as the relation of regional ΔZ/total ΔZ expressed in percentage and used to estimated regional tidal volume VTr = regional ΔZ/total ΔZ x total V_T_).
- Regional lung compliance (C_Z_) was calculated as regional VTr /ΔP.
- The change in lung aeration was estimated by the change in EELI [ΔEELI x (V_T_/ΔZ)].

Lung perfusion was recorded during an expiratory pause by injecting a 10-ml bolus of 7.5% hypertonic saline solution into a central venous catheter. The perfusion maps were done with a specific and validated Enlight 1800 EIT tool [4].

Ventilation and perfusion maps were segmented into regions of interest (ROI) as follows [5]:

- To compare changes during supine position steps, the lungs were segmented into two equally sized ROIs: ventral (Upper lung or anterior half) and dorsal (Lower lung or posterior half).
- To compare changes from supine to lateral position (right or left decubitus), the lungs were also segmented into four ROIs or quadrants according to the new situation when lateralized: ventral non-dependent, dorsal non-dependent, dorsal dependent, and ventral dependent.

**Lung ultrasound**

LUS was performed with the ultrasound MyLab Gold 25 device (Esaote, Genova, Italy) using a macro-convex probe of 3-5 MHz. Each hemithorax was segmented into six regions using three longitudinal lines (anterior, parasternal, and posterior axillary) and one axial line at the 5th intercostal space. The LUS aeration score with the information from the 12 lung regions was calculated. The score is based on four patterns: 1) normal aeration (N) depicted by normal LUS images with lung sliding (i.e., movement of pleura during breathing) and A-lines (i.e., artifacts observed as hyperechoic horizontal lines repeated at regular intervals). 2) Presence of few B lines (B1), which are vertical dynamic lines, originating from the pleural line reaching the lowest edge of the screen. 3) Multiple coalescent B-lines (B2). 4) Complete loss of aeration with the presence of consolidations (C). For each thoracic area, the score was allocated as follow: N = 0, B1 = 1, B2 = 2 and C = 3 points. The LUS aeration score was calculated by the sum of points, ranging from normal aeration (0 point) to the worst possible aeration (18 points for each hemithorax) [6].

The degree of juxta-pleural consolidation was divided into four grades and scored between 0 and 3: (0) no consolidation; (1) minimal juxta-pleural consolidation; (2) small-sized consolidation; (3) large-sized consolidation. The consolidation score was calculated by the sum of points from the 12 lung regions [7].

**Blood gas analysis**

ABL800FLEX (Radiometer, Copenhagen, Denmark) was used for arterial gas analysis.

The end-tidal partial pressure of carbon dioxide (PETCO_2_) was measured using a mainstream infrared CO_2_ sensor capnostat-3® (Philips Respironics, Philadelphia, PA) integrated within the ventilator, and the arterial-to-end-tidal difference of CO_2_ (Pa-ETCO_2_) was calculated.

**Protocol**

Patients were studied in five body positions in a sequential order 30 minutes each:

- Supine-1 (baseline);
- Lateral-1 (the less ventilated lung evaluated by EIT was positioned up);
- Supine-2 (after first lateral position);
- Lateral-2 (the contralateral lung was positioned up);
- Supine-3 (after second lateral position).

Lateral positioning was performed using a custom-made support cushion lined with special foam to prevent pressure injuries with a fixed inclination of 30° for better patient tolerance and positioning consistency as well as a better quality of the EIT images (Figure E1).

At the end of each step (30 min), the following measurements were recorded in this sequence:

- Blood gas analysis
- Respiratory mechanics
- EIT measurements
- Lung ultrasound (in lateral steps, only was examined the non-dependent lung)

**REFERENCES**

1. ARDS Definition Task Force, Ranieri VM, Rubenfeld GD, Thompson BT, Ferguson ND, Caldwell E, Fan E, Camporota L, Slutsky AS, (2012) Acute respiratory distress syndrome: the Berlin Definition. JAMA 307: 2526-2533. https://doi.org/10.1001/jama.2012.5669

2. Chen L, Del Sorbo L, Grieco DL, Junhasavasdikul D, Rittayamai N, Soliman I, Sklar MC, Rauseo M, Ferguson ND, Fan E, Richard JM, Brochard L, (2020) Potential for Lung Recruitment Estimated by the Recruitment-to-Inflation Ratio in Acute Respiratory Distress Syndrome. A Clinical Trial. Am J Respir Crit Care Med 201: 178-187. https://doi.org/10.1164/rccm.201902-0334OC

3. Baydur A, Behrakis PK, Zin WA, Jaeger M, Milic-Emili J, (1982) A simple method for assessing the validity of the esophageal balloon technique. Am Rev Respir Dis 126: 788-791. https://doi.org/10.1164/arrd.1982.126.5.788

4. Borges JB, Suarez-Sipmann F, Bohm SH, Tusman G, Melo A, Maripuu E, Sandstrom M, Park M, Costa EL, Hedenstierna G, Amato M, (2012) Regional lung perfusion estimated by electrical impedance tomography in a piglet model of lung collapse. J Appl Physiol (1985) 112: 225-236. https://doi.org/10.1152/japplphysiol.01090.2010

5. Frerichs I, Amato MB, van Kaam AH, Tingay DG, Zhao Z, Grychtol B, Bodenstein M, Gagnon H, Bohm SH, Teschner E, Stenqvist O, Mauri T, Torsani V, Camporota L, Schibler A, Wolf GK, Gommers D, Leonhardt S, Adler A, group Ts, (2017) Chest electrical impedance tomography examination, data analysis, terminology, clinical use and recommendations: consensus statement of the TRanslational EIT developmeNt stuDy group. Thorax 72: 83-93. https://doi.org/10.1136/thoraxjnl-2016-208357

6. Bouhemad B, Brisson H, Le-Guen M, Arbelot C, Lu Q, Rouby JJ, (2011) Bedside ultrasound assessment of positive end-expiratory pressure-induced lung recruitment. Am J Respir Crit Care Med 183: 341-347. https://doi.org/10.1164/rccm.201003-0369OC

7. Monastesse A, Girard F, Massicotte N, Chartrand-Lefebvre C, Girard M, (2017) Lung Ultrasonography for the Assessment of Perioperative Atelectasis: A Pilot Feasibility Study. Anesth Analg 124: 494-504. https://doi.org/10.1213/ANE.0000000000001603

**Figure S1.** Lateral positioning of the patient on the right side using a support with 30° angulation.

**Table S1. Lung ultrasound and EIT parameters during supine position steps**

|  | **Position** | | | | |
| --- | --- | --- | --- | --- | --- |
|  | **Supine-1** | **Supine-2** | **Supine-3** | **N** | **p** |
| **Lung ultrasound** |  |  |  |  |  |
| LUS score | 14.9 ± 4.2 |  | 12.6 ± 5.2 | 15 | 0,07 |
| Consolidation score | 5 (4-5) |  | 2 (1-4) | 15 | < 0.01 |
| **EIT parameters** |  |  |  |  |  |
| C_Z_ ventral region, mL/cmH_2_O | 11.5 ± 2.9 | 12.5 ± 2.8 | 12.9 ± 3.0 | 15 | < 0.01 |
| C_Z_ dorsal region, mL/cmH_2_O | 17.1 ± 6.9 | 18.1 ± 6.6 | 18.8 ± 7.1 | 15 | < 0.01 |
| Ventral ΔEELI, mL | 0 | -121 ± 157 | -218 ± 205 | 15 | < 0.01 |
| Dorsal ΔEELI, mL | 0 | 338 ± 350 | 192 ± 475 | 15 | 0.02 |
| Ventral fraction of tidal ventilation, (%) | 41.6 ± 7.9 | 41.8 ± 7.3 | 41.9 ± 8.6 | 15 | 0.92 |
| Dorsal fraction of tidal ventilation, (%) | 58.4 ± 7.9 | 58.2 ± 7.3 | 58.1 ± 8.6 | 15 | 0.92 |
| Ventral fraction of perfusion, (%) | 35.2 ± 7.3 |  | 33.7 ± 7.2 | 15 | 0.01 |
| Dorsal fraction of perfusion, (%) | 64.8 ± 7.3 |  | 66.3 ± 7.2 | 15 | 0.01 |

Abbreviations: LUS score: Lung ultrasound score; Cz: Regional compliance; ΔEELI: Changes in end-expiratory lung impedance; N: number of patients.

Continuous variables are shown as mean ± SD or median (IQR) based on their distribution.

Mixed model was used to compare the periods in the supine position.

Paired t-test or Wilcoxon signed-rank test was used to compare two measurements based on their distribution.

**Table S2.** Changes of parameters during lateral positioning

|  | **First Lateral positioning** | | | | **Second lateral positioning** | | | |
| --- | --- | --- | --- | --- | --- | --- | --- | --- |
|  | **Supine** | **Lateral** | **N** | **p** | **Supine** | **Lateral** | **N** | **p** |
| **Respiratory parameters** |  |  |  |  |  |  |  |  |
| Driving airway pressure, cmH_2_O | 12.5 ± 2.4 | 13.2 ± 2.5 | 15 | 0,02 | 11.6 ± 2.2 | 13.6 ± 3.1 | 15 | < 0.01 |
| Respiratory system compliance, mL/cmH_2_O | 28.5 ± 8.5 | 26.8 ± 7.8 | 15 | 0,01 | 30.6 ± 8.1 | 26.3 ± 8.1 | 15 | < 0.01 |
| Plateau airway pressure, cmH_2_O | 26.8 ± 2.9 | 27.5 ± 3.0 | 15 | 0,03 | 25.9 ± 2.7 | 27.9 ± 2.8 | 15 | < 0.01 |
| Total PEEP, cmH_2_O | 15.5  (12.5-15.8) | 15.5  (12.4-15.8) | 15 | 0,64 | 15.4  (12.4-16.1) | 15.6  (12.4-15.7) | 15 | 0,3 |
| Resistance, cmH_2_O/L/min | 10.9  (10-12) | 11.5  (10.8-12.5) | 15 | 0,03 | 11.3  (10.5-12.4) | 11.5  (10.6-12.4) | 15 | 0,76 |
| Driving transpulmonary pressure, cmH_2_O | 10.9 ± 2.5 | 11 ± 2.7 | 14 | 0,78 | 10.2 ± 2.3 | 11.4 ± 3.5 | 14 | 0,03 |
| Lung compliance, ml/cmH_2_O | 33.8 ± 10.9 | 33.3 ± 10.1 | 14 | 0,67 | 35.7 ± 10.5 | 33.1 ± 11.7 | 14 | 0,06 |
| Inspiratory transpulmonary pressure, cmH_2_O | 15.9 ± 4.7 | 16.7 ± 5.0 | 14 | 0,42 | 15.1 ± 4.5 | 15.9 ± 6.0 | 14 | 0,6 |
| Expiratory transpulmonary pressure, cmH_2_O | 5.0 ± 3.6 | 5.7 ± 4.1 | 14 | 0,45 | 4.9 ± 4.1 | 4.4 ± 4.3 | 14 | 0,76 |
| Chest wall compliance, mL/cmH_2_O | 240 ± 59 | 187 ± 51 | 14 | < 0.01 | 248 ± 90 | 172 ± 61 | 14 | < 0.01 |
| PaO_2_/FIO_2_, mmHg | 137.5 ± 36.3 | 154.3 ± 45.2 | 15 | 0,08 | 158.4 ± 35.9 | 154.8 ± 56.8 | 15 | 0,65 |
| PaCO_2_, mmHg | 63 (58-75) | 66 (55-81) | 15 | 0,68 | 63 (52-71) | 66 (57-75) | 15 | < 0.01 |
| pH | 7.28 ± 0.1 | 7.27 ± 0.1 | 15 | 0,22 | 7.28 ± 0.1 | 7.26 ± 0.1 | 15 | < 0.01 |
| SpO_2_, (%) | 96 (95-98) | 97 (96-98) | 15 | 0,26 | 98 (96-99) | 98 (95-100) | 15 | 0,1 |
| ETCO_2_, (mmHg) | 52.5 ± 10.7 | 51.2 ± 10.6 | 15 | 0,18 | 50.5 ± 10.6 | 51.5 ± 9.6 | 15 | 0,28 |
| PaCO_2_ - ETCO_2_, (mmHg) | 13.3  (7.2-19.4) | 15.3  (7.7-28.5) | 15 | 0,17 | 12.4  (6.2-17.2) | 16.4  (7.4-21) | 15 | 0,03 |
| **Hemodynamic parameters** |  |  |  |  |  |  |  |  |
| Heart rate, beats/minute | 92 ± 18 | 94 ± 19 | 15 | 0,54 | 90 ± 17 | 93 ± 18 | 15 | 0,19 |
| Systolic arterial pressure, mmHg | 127 ± 16 | 121 ± 20 | 15 | 0,24 | 124 ± 19 | 118 ± 21 | 15 | 0,24 |
| Diastolic arterial pressure, mmHg | 68 ± 8 | 69 ± 7 | 15 | 0,64 | 68 ± 11 | 64 ± 10 | 15 | 0,21 |
| Mean arterial pressure, mmHg | 88 ± 10 | 86 ± 11 | 15 | 0,67 | 86 ± 13 | 83 ± 12 | 15 | 0,32 |
| **Lung ultrasound (non-dependent lung)** |  |  |  |  |  |  |  |  |
| LUS score | 7.9 ± 2.3 | 4.9 ± 3 | 14 | < 0.01 | 6.1 ± 2.9 | 3.9 ± 2.3 | 14 | < 0.01 |
| Consolidation score | 2 (1-3.5) | 0 (0-0.25) | 14 | < 0.01 | 3 (2.5-5) | 1 (0-3) | 14 | < 0.01 |
| **EIT parameters** |  |  |  |  |  |  |  |  |
| Fraction of tidal ventilation, non-dependent lung (%) | 42.5 ± 5.6 | 29.5 ± 10.1 | 15 | < 0.01 | 55 ± 7.4 | 39.4 ± 8.6 | 15 | < 0.01 |
| Fraction of tidal ventilation, dependent lung (%) | 57.5 ± 5.6 | 70.5 ± 10.1 | 15 | < 0.01 | 45 ± 7.4 | 60.6 ± 8.7 | 15 | < 0.01 |
| Fraction of perfusion, non-dependent lung (%) | 45.2 ± 8.6 | 44.6 ± 9.6 | 14 | 0,76 | 54.5± 8.4 | 52.9 ± 7 | 14 | 0,36 |
| Fraction of perfusion, dependent lung (%) | 54.8 ± 8.6 | 55.4 ± 9.6 | 14 | 0,76 | 45.5± 8.4 | 47.1 ± 7 | 14 | 0,36 |

Abbreviations: PEEP= Positive end-expiratory pressure; PaO_2_/FIO_2_= partial pressure of oxygen in arterial blood / inspired oxygen fraction ratio; PaCO_2_= partial pressure of carbon dioxide in arterial blood; SpO_2_= oxygen saturation; ETCO_2_= End-tidal CO_2_; n: number of patients.

Continuous variables are shown as mean ± SD or median (IQR) based on their distribution.

To evaluate the variables during two periods of time (supine vs. lateral) was used a paired t-test or Wilcoxon signed-rank test based on their distribution.

**Table S3.** Changes of parameters after applying a postural recruitment maneuver according to recruitability, low vs high, based on recruitment-to-inflation ratio

| **Parameters** | **Low recruitability group** | | | | | **High recruitability group** | | | | |
| --- | --- | --- | --- | --- | --- | --- | --- | --- | --- | --- |
|  | **Supine-1** | **Supine-2** | **Supine-3** | **N** | **P** | **Supine-1** | **Supine-2** | **Supine-3** | **N** | **P** |
| **Respiratory parameters** |  |  |  |  |  |  |  |  |  |  |
| Driving airway pressure, cmH_2_O | 12.5 ± 2.7 | 11.8 ± 2.4 | 11.4 ± 2.1 | 7 | 0.14 | 12.5 ± 2.4 | 11.4 ± 2.2 | 11.0 ± 2.2 | 8 | < 0.01 |
| Respiratory system compliance, mL/cmH_2_O | 27.6 ± 10.7 | 28.8 ± 9 | 29.6 ± 9.3 | 7 | 0.26 | 29.2 ± 6.8 | 32.2 ± 7.6 | 33.4 ± 7.7 | 8 | < 0.01 |
| Driving transpulmonary pressure, cmH_2_O | 10.7 ± 3.0 | 10.6 ± 2.4 | 9.9 ± 2.1 | 6 | 0.19 | 11.0 ± 2.4 | 9.9 ± 2.2 | 9.5 ± 2.2 | 7 | < 0.01 |
| Lung compliance, ml/cmH_2_O | 34.3 ± 14.6 | 33.5 ± 11.9 | 35.6 ± 12.1 | 6 | 0.44 | 33.4 ± 8.3 | 37.4 ± 9.7 | 38.9 ± 9.8 | 7 | < 0.01 |
| PaO_2_/FIO_2_, mmHg | 119 ± 26 | 137 ± 26 | 141 ± 38 | 7 | 0.08 | 154 ± 38 | 178 ± 33 | 184 ± 45 | 8 | 0.01 |
| **Lung ultrasound** |  |  |  |  |  |  |  |  |  |  |
| LUS score (*) | 15 ± 3.4 |  | 14.4 ± 5.5 | 7 | 0.8 | 14.9 ± 5 |  | 11 ± 4.7 | 8 | 0.01 |
| Consolidation score (**) | 5 (4-5) |  | 3 (0-4) | 7 | 0.27 | 4.5 (3.25-6.5) |  | 2 (1.25-4.5) | 8 | 0.016 |
| **EIT parameters** |  |  |  |  |  |  |  |  |  |  |
| C_Z_ ventral region, mL/cmH_2_O | 11.9 ± 4 | 12.8 ± 4 | 13.5 ± 4.3 | 7 | 0.086 | 11.1 ± 1.7 | 12.2 ± 1.3 | 12.3 ± 1.3 | 8 | < 0.01 |
| C_Z_ dorsal region, mL/cmH_2_O | 15.8 ± 7 | 16.1 ± 5.5 | 16 ± 5.7 | 7 | 0.94 | 18.1 ± 7 | 19.9 ± 7.2 | 21.1 ± 7.7 | 8 | < 0.01 |
| Ventral ΔEELI, mL | 0 | -44 ± 166 | -132 ± 241 | 7 | 0.217 | 0 | -106 ± 192 | -226 ± 197 | 8 | 0.016 |
| Dorsal ΔEELI, mL | 0 | 285 ± 268 | 240 ± 308 | 7 | 0.096 | 0 | 311 ± 379 | 80 ± 533 | 8 | 0.147 |

Abbreviations: PaO2/FIO2= partial pressure of oxygen in arterial blood / inspired oxygen fraction ratio; LUS score=Lung ultrasound score; Cz= Regional compliance; ΔEELI: Change in end-expiratory lung impedance; n: number of patients.

Continuous variables are shown as mean ± SD or median (IQR) based on their distribution. Mixed model was used to compare the periods in supine position.

(*) Paired t test was used for the analysis of LUS score and (**) Wilcoxon signed-rank test for the consolidation score.

**Figure S2.** Illustrative lung ultrasound images from two patients after a postural recruitment maneuver.

In the upper panel (A) are shown ultrasound images of a responder patient with re-aeration of consolidates in the right and left posterior regions of the lung after each lateral positioning. In the lower panel (B), ultrasound images of a non-responder patient show increase in consolidates on the right and left posterior regions of the lung after each lateral positioning.

**Figure S3. Changes in the EELI of each lung according to lateral positioning sequence.**

In the upper section (A) are shown the changes in EELI of both lungs through lateral positioning sequence. Lung Up-first (in red) represents the changes in the lung that was positioned upwards first (L1 during the first lateral position). Lung Up-last (in blue) describes the changes in the lung that was positioned upwards last (L2 during the second lateral position).

In the lower section, both lungs are individualized (B and C), and it is shown a marked increase in the EELI of the non-dependent lung (uppermost) without change in EELI of the dependent lung (lowermost) during both lateral positions.

Δ EELI (mL): End-expiratory lung impedance change. Data are shown as mean ± SEM. For the statistical analysis, a mixed model was used; all posthoc comparisons were adjusted through Sidak correction.

**Figure S4. Changes in the regional compliance of each lung according to lateral positioning sequence.**

Lung Up-first (red circles) represents the changes in the regional compliance (C_z_) of the lung that was positioned upwards first (L1 during the first lateral position). Lung Up-last (blue circles) represents the changes in the regional compliance of the lung that was positioned upwards last (L2 during the second lateral position).

After the application of the postural recruitment maneuver, once the patient returns to supine, the regional compliance of the lung that was positioned upwards during **l**ateral positioning increased while the lung that was positioned downwards maintained its compliance. At the end of the **l**ateral positioning sequence, both lungs improved their compliance with respect to baseline (supine 1).

C_z_: Regional compliance. Data are shown as mean ± SEM. For the statistical analysis, a mixed model was used; all posthoc comparisons were adjusted through Sidak correction.

**Figure S5.** **Representative patient illustrating the response in respiratory mechanics and oxygenation after the application of a postural recruitment maneuver.**

The figures at the top (ventilation map) show the five body positions of the protocol in sequential order. The figures of the middle part compare the variation in regional compliance between supine-2 and supine-3 steps with respect to baseline (supine-1). Once the patient returns to the supine position, there is an increase in regional compliance (represented in green) of the lung previously positioned upwards during lateral positioning. At the end of the lateral positioning sequence (supine-3), the respiratory system compliance increased, the driving pressure decreased, and oxygenation improved (lower part).

C_Z_: Regional compliance; R/L: segmenting the lungs into right and left; C_RS_: Respiratory system compliance; DP: Driving pressure; PaO_2_/FIO_2_= partial pressure of oxygen in arterial blood / inspired oxygen fraction ratio. Data are shown as mean ± SEM
